# Species recognition limits mating between hybridizing ant species

**DOI:** 10.1111/evo.14566

**Published:** 2022-07-19

**Authors:** Pierre Blacher, Sacha Zahnd, Jessica Purcell, Amaury Avril, Thalita Oliveira Honorato, Gaëlle Bailat‐Rosset, Davide Staedler, Alan Brelsford, Michel Chapuisat

**Affiliations:** ^1^ Departement of Ecology and Evolution University of Lausanne Lausanne CH‐1015 Switzerland; ^2^ Department of Entomology University of California Riverside California 92521 USA; ^3^ Scitec Research SA Lausanne CH‐1007 Switzerland; ^4^ Department of Biomedical Sciences University of Lausanne Lausanne CH‐1011 Switzerland; ^5^ Department of Biology University of California Riverside California 92521 USA

**Keywords:** Assortative mating, hybrid zone, hydrocarbon cues, *Formica* ants, speciation, species recognition

## Abstract

Identifying mechanisms limiting hybridization is a central goal of speciation research. Here, we studied premating and postmating barriers to hybridization between two ant species, *Formica selysi* and *Formica cinerea*. These species hybridize in the Rhône valley in Switzerland, where they form a mosaic hybrid zone, with limited introgression from *F. selysi* into *F. cinerea*. There was no sign of temporal isolation between the two species in the production of queens and males. With choice experiments, we showed that queens and males strongly prefer to mate with conspecifics. Yet, we did not detect postmating barriers caused by genetic incompatibilities. Specifically, hybrids of all sexes and castes were found in the field and F1 hybrid workers did not show reduced viability compared to nonhybrid workers. To gain insights into the cues involved in species recognition, we analyzed the cuticular hydrocarbons (CHCs) of queens, males, and workers and staged dyadic encounters between workers. CHC profiles differed markedly between species, but were similar in *F. cinerea* and hybrids. Accordingly, workers also discriminated species, but they did not discriminate *F. cinerea* and hybrids. We discuss how the CHC‐based recognition system of ants may facilitate the establishment of premating barriers to hybridization, independent of hybridization costs.

Hybridization and gene flow between species play key roles in fundamental evolutionary processes such as adaptation and speciation. The widespread application of genome sequencing has led to the realization that hybridization is more common than previously thought (Mallet 2005, 2007; Ellstrand and Rieseberg 2016). By bringing together independently evolving genomes, hybridization often negatively affects the fitness of individuals, a phenomenon known as “hybrid breakdown” (Coyne and Orr 1998; Burke and Arnold 2001; Abbott et al. 2013). Such costs might play a crucial role in the early evolution and later maintenance of species, by selecting for mechanisms limiting interspecific gene flow.

Hybridization can be limited through multiple reproductive isolating barriers occurring before or after mating (Coyne et al. 2004). Premating barriers to hybridization include spatial isolation, temporal isolation, and mate choice. Postmating barriers to introgression comprise hybrid inviability and hybrid sterility. The various mechanisms influence each other in a feedback loop (i.e., reinforcement; Servedio and Noor 2003; Coyne et al. 2004). In particular, low hybrid fitness selects for assortative mating (Coyne and Orr 1998; Burke and Arnold 2001; Coyne et al. 2004; Chatfield et al. 2010; Abbott et al. 2013; Shizuka and Hudson 2020). However, species recognition and assortative mating can also evolve independently of hybridization costs, due to drift or local adaptation (Hollander et al. 2005), or as a by‐product of sexual or kin selection (Gleason and Ritchie 1998; Servedio 2016). Determining the impact and causal relationship of pre‐ and postmating isolation mechanisms on hybridization patterns is important to understanding the evolutionary processes limiting hybridization and leading to speciation (Irwin 2020).

The speciation process has received surprisingly little attention in the social Hymenoptera. Yet, social Hymenoptera present several characteristics that make them valuable models for investigating hybridization patterns (Seifert 1999; Feldhaar et al. 2008; Kulmuni et al. 2010; Beresford et al. 2017). Because of their male‐haploid female‐diploid sex determination system, males are expected to suffer higher fitness consequences of hybridization, as all introgressed alleles are exposed to selection. This fitness asymmetry can lead to hybrid zones composed of hybrid females and nonhybrid males (Kulmuni and Pamilo 2014). Furthermore, in many species females predominantly mate with a single male at the beginning of their adult lives. This lifelong commitment between partners exposes them to large hybridization costs, as potential genetic incompatibilities between mates cannot be mitigated by remating (Feldhaar et al. 2008). Fertile hybrid queens and males are generally rare in the social Hymenoptera, which suggests that hybridization costs are high (Feldhaar et al. 2008; Kulmuni et al. 2010). High hybridization costs may favor the evolution of effective premating barriers. Yet, hybridization is common in several ant lineages, although its directionality (unidirectional or reciprocal) and frequency greatly vary between species (Feldhaar et al. 2008). In some systems, hybridization results in hybridogenesis, whereby workers are systematically produced by hybridization between two lineages (Lavanchy and Schwander 2019). In view of this diversity of outcomes, the relationship between hybridization costs, species recognition, and reproductive isolation deserves further investigation.

The advanced recognition system of social Hymenoptera could facilitate the evolution of premating barriers. Social insects have developed effective recognition abilities to deal with territorial competition, nest defense, and mate choice (Ayasse et al. 2001; d'Ettorre and Lenoir 2010; Leonhardt et al. 2016). This recognition system is based on chemicals, mostly hydrocarbons, present on the ant cuticles (Ayasse et al. 2001; Howard and Blomquist 2005). Because cuticular hydrocarbons (CHCs) have a strong genetic component, genetic drift or local adaption may lead to divergent CHC profiles between species (Gleason et al. 2009; Schwander et al. 2013; Dembeck et al. 2015), which in turn may entail species discrimination and lead to assortative mating, even when hybridization costs are absent (Drescher et al. 2010; Xue et al. 2018). Furthermore, CHCs are likely to influence the behavioral interactions of hybrids with members of their parent species. Investigating the CHC profiles and mutual behavior of hybrids and parent species can thus help to explain the dynamics of hybrid zones in social insects.

The discovery of a mosaic hybrid zone between the ant species *Formica selysi* and *Formica cinerea* (Purcell et al. 2016) set an ideal foundation for the study of barriers to hybridization in ants. *Formica selysi* and *F. cinerea* are socially polymorphic species: colonies can be headed by a single queen (monogyne) or by multiple queens (polygyne) (Goropashnaya et al. 2001; Chapuisat et al. 2004; Purcell and Chapuisat 2013). *Formica cinerea* is broadly distributed across Europe, whereas *F. selysi* is mainly present in the Alps and the Pyrenees. Both species occupy sparsely vegetated, sunny, sandy areas. *Formica selysi* is particularly abundant near streams and rivers. Hybridization was reported along the Rhône valley in Switzerland (Purcell et al. 2016). Interestingly, hybrids were relatively rare, amounting to 20% of the workers genotyped. Most hybrid workers had a genetic background skewed toward *F. cinerea*. These genomic data suggest that some mechanisms restrict gene flow between species, but also that hybrids are fertile and mainly backcross with *F. cinerea* (Purcell et al. 2016). Preliminary assessment of the CHC profiles and behavior of workers from the two species also suggested that species recognition mechanisms might play a role in this asymmetric hybridization (Purcell et al. 2016), possibly helping to stabilize the mosaic hybrid zone (M'Gonigle and FitzJohn 2010). This prompted us to investigate the temporal, behavioral, and genetic barriers to hybridization between the two species and to further study the putative roles of species discrimination and CHC recognition cues in restricting between‐species gene flow.

To better understand the maintenance and dynamics of this hybrid zone, we investigated two potential premating barriers, temporal segregation and assortative mate preference, and one potential postmating barrier, the reduced viability of hybrid offspring caused by genetic incompatibilities. We provide evidence that assortative mate preference and species‐specific CHC cues occur in both species, and we discuss how asymmetries in CHC resemblance and discrimination might bias gene flow between the two species.

## Materials and Methods

### GENERAL EXPERIMENTAL APPROACH, SAMPLING, AND GENOTYPING

We assessed temporal isolation between *F. selysi* and *F. cinerea* by monitoring the timing of production of winged queens and males. We then staged controlled mate choice experiments to examine whether queens and males preferentially mate with partners of their own species. To study genetic incompatibilities between species, we monitored brood production by queens mated with conspecifics or heterospecifics. In addition, we checked whether viable hybrid workers, queens, and males occur in the field. Finally, we examined whether workers behaviorally discriminate conspecifics, hybrids, and heterospecifics, and studied in workers, queens, and males the CHCs likely involved in species recognition.

Field sampling and monitoring took place in nine populations from central Valais, Switzerland (Blitzingen, Branson, Derborence, Finges, Les Barges, Riddes, Saillon, Sion, and Ulrichen). These populations harbor pure *F. selysi*, pure *F. cinerea*, and/or hybrid individuals, in varying proportions (Purcell et al. 2016). Each nest was covered with a numbered flat stone to facilitate nest identification, monitoring over time, and sample collection. We sampled winged queens, males, and workers over 6 years (2014–2018 and 2021).

We identified the species and hybrids by genotyping diagnostic SNPs of at least two workers per colony. DNA was extracted from one leg crushed in 100 μL of ddH_2_0 with 10% Chelex^©^ and 5 μL of proteinase K (Qiagen, 20 mg/mL), incubated at 55°C for 40 min, followed by 100°C for 20 min. With a PCR‐RFLP assay, we genotyped one mitochondrial and three nuclear SNPs presenting fixed differences between *F. selysi* and *F. cinerea* (Purcell et al. 2016). Individuals were classified as hybrids when they were heterozygous at one or more SNPs or had a combination of homozygous SNPs specific to *F. selysi* and *F. cinerea*. This design is very powerful to detect F1 hybrids (100% of detection) and first backcrosses (e.g., colonies of F2 hybrids are detected with a probability of 99.6% when genotyping two workers). For behavioral assays with workers and chemical analyses, we calculated for each colony a hybrid index (HI) based on the following scores for each nuclear SNP: homozygous *F. cinerea* = 0, homozygous *F. selysi* = 1, and heterozygous = 0.5. The HI of a colony was calculated as the average score of the three nuclear SNPs across three workers per colony and ranged from 0 (*F. cinerea* colony) to 1 (*F. selysi* colony).

### TEMPORAL ISOLATION

To assess whether the timing of queen and male production constitutes a premating barrier to hybridization, we monitored the production of winged queens (i.e., unmated females destined to become queens) and males in 36 colonies of pure *F. selysi* and 16 colonies of pure *F. cinerea*, as inferred by genotyping three workers per colony. The colonies were located in three populations harboring both species (Branson, Riddes, and Saillon; Purcell et al. 2016). We visited each colony on a weekly basis, in June and July 2014, and lifted the stones covering the colonies to record the presence or absence of winged queens or males inside the colony.

### MATE CHOICE

To assess whether queens and males prefer to mate with partners of their own species, we performed mate choice experiments. We sampled winged unmated queens, males, and workers from 146 colonies in nine populations (see above) during summer 2014, 2015, 2016, and 2018. Colony fragments were transferred to plastic boxes (15.5 × 13.5 × 5.5 cm) lined with fluon and containing a glass tube (length = 16 cm; ø = 5 mm) one‐third filled with water. They were maintained in the laboratory in a 12:12 dark:light cycle, at 24°C, and under a relative humidity of 50%. The ants were provided with water and sugar‐egg jelly ad libitum. We kept the unmated queens and males in separate plastic boxes, to prevent intranidal mating. We genotyped three workers per colony, which allowed us to identify 92 pure *F. selysi*, 44 pure *F. cinerea*, and 10 hybrid colonies that produced queens and/or males. We retained the pure *F. selysi* and pure *F. cinerea* colonies for mate choice and genetic incompatibility experiments.

Mate choice experiments were performed in controlled conditions, following the procedure described in Avril et al. (2019). In each assay, one unmated female (queen), either *F. cinerea* or *F. selysi*, was placed with four color‐marked unmated males, two per species, in a mating arena consisting of a box covered by a net (35 × 22 × 15 cm). The female and the males originated from different colonies and, whenever possible, from different populations. The observers were kept blind with respect to the species involved. The mating boxes were placed outdoors, on sunny mornings. We observed the queens and males for up to 120 mins and collected all mating pairs. We isolated the mated queens in glass tubes one‐third filled with water.

### GENETIC INCOMPATIBILITIES BETWEEN SPECIES

To test for genetic incompatibilities between species, we monitored survival and brood production of *F. selysi* and *F. cinerea* queens mated either to males of their own species or to males of the other species. The glass tubes containing the mated queens were covered with aluminum foil and placed in the dark to mimic natural conditions of independent colony founding. We monitored each queen individually two to four times a week during six consecutive weeks, recording (i) whether the queens were alive, (ii) whether they produced brood, and (iii) their number of offspring. We included in this experiment all queens that mated in the mate choice experiment, plus additional queens that mated without choice (i.e., were presented to males of only one species, in the same experimental conditions; Avril et al. 2019). Details on queen samples are given in Table S1.

We also assessed if genetic incompatibilities affected the production of hybrid winged queens and males by monitoring their production in hybrid field colonies (determined via genotyping workers, see above). In June and July, over 3 years (2014, 2015, and 2018), we visited once or twice hybrid colonies from three populations and recorded the number of winged queens or males present (Table S2; Purcell et al. 2016). We genotyped most of the winged queens and males to confirm their hybrid genetic background.

### DYADIC ENCOUNTERS

To investigate whether workers also recognize and behaviorally discriminate conspecifics, heterospecifics, and hybrids, we performed dyadic encounters. We collected workers from the Branson population in October 2017. We genotyped three workers per colony and retained for the experiments three pure *F. selysi* colonies, six pure *F. cinerea* colonies, and eight hybrid colonies. We housed the nestmate workers in separate plastic boxes (15.5 × 13.5 × 5.5 cm) lined with fluon and containing a glass tube (length = 16 cm; ø = 5 mm) one‐third filled with water. The workers were maintained at 25°C, with a humidity level of 70%, in a 12:12 h light:dark cycle. They were provided with sugar‐egg jelly twice a week.

Workers were paint‐marked 48 h before the assays, using color combinations allowing for individual identification. We tested the six following dyads of workers: *F. cinerea* versus *F. cinerea* (*n* = 23), *F. selysi* versus *F. selysi* (*n* = 20), Hybrid versus Hybrid (*n* = 16), *F. cinerea* versus *F. selysi* (*n* = 33), Hybrid versus *F. cinerea* (*n* = 31), and Hybrid versus *F. selysi* (*n* = 27). All tested workers within dyads were non‐nestmates. The dyadic encounters took place in a neutral arena consisting of a 6‐cm Petri dish side‐lined with fluon, with a filter paper on the bottom. For each assay, two workers were transferred to separate compartments of the neutral arena. After 1 min, the partitions were removed to allow workers to interact freely. We video‐recorded the behavior of workers for 3 min. The tested workers were freeze‐killed after the assay, stored in glass vials at −20°C, and the filter paper was replaced to remove odors. The order of assays was randomized among the six types of dyadic encounters.

We measured the occurrence and duration of each behavior for each worker involved in a dyadic encounter with the software BORIS version 5.1.0 (Friard and Gamba 2016). The scorer of the videos was kept blind to the species of the tested ants. We calculated an aggression index (AI) based on the following scores for each behavior (adapted from Hefetz et al. 1996; Errard and Hefetz 1997): 0, antennation (neutral interaction); 1, mandible opening (threat); 2, biting (moderately aggressive interaction); 3, biting with acid spraying (highly aggressive interaction). The overall aggression exhibited by each worker (AI) was calculated as follows:

$$\mathrm{AI}=\frac{\sum_{i=1}^{n}{\mathrm{AI}}_{i}\times t_{i}}{T},$$

where AI*_i_* represents the score of the interaction *i*, *t_i_* the duration of each interaction, and *T* the total interaction time, defined as the sum of durations of all interactions.

### SPECIES RECOGNITION: GCMS ANALYSIS OF CHCs

To get insights into the cues involved in species recognition, we performed GCMS analyses of CHCs. We analyzed the CHCs of workers (two replicates per colony) from the three *F. selysi*, six *F. cinerea*, and eight hybrid colonies used for dyadic encounters in 2017. We also analyzed the CHCs of workers, males, and winged queens from 12 *F. selysi* and 12 *F. cinerea* colonies collected in 2021 in the same Branson population. The cuticular compounds were extracted by immersing three workers, one male, or one winged queen, respectively, in 320 μL of hexane (99% pure) for 15 min. The solvent extract was transferred to a new vial, where it evaporated. Each extract was then dissolved again in 30 μL of hexane, complemented with 10 ng/μL of eicosane (*n*C_20_; not present in *F. selysi* and *F. cinerea*), which served as internal standard. A total of 2 μL of each extract was injected into an Agilent gas chromatograph tandem mass spectrometer (GC‐MSMS Agilent 7010, USA) equipped with an Agilent 19091S‐433UI HP5‐MS column. The carrier gas (helium) flow rate was set at 3 mL/min. Injection temperature was set to 250°C in splitless mode. The temperature ramp was set at 70°C and increased to 300°C at 3°C/min, then maintained at 300°C for 3 min (total run time: 67.67 min). The analysis was carried out in a full scan acquisition mode (50–500 amu).

Peak areas were integrated with OPENChrome software version 1.4.0. We removed small peaks and erratic compounds by excluding peaks whose relative abundance amounted to less than 0.2%, and/or that were detected in less than half of the chromatograms of each caste and sex of each species (see Blacher et al. 2013). Contaminants were identified and excluded on the basis of mass spectra. The relative abundance of the 74 peaks retained for analysis was then re‐calculated (Table S3). The compounds were identified using mass spectra, their retention times, and published literature on *F. selysi* (Meunier et al. 2011).

### STATISTICAL ANALYSIS

All analyses were conducted using R version 4.1.2 (R Core Team 2021). Models were tested using the “glmmTMB” package (Brooks et al. 2017) and regression assumptions were evaluated using diagnostic plots with the package DHARMa (Hartig 2020). Nonsignificant interaction terms were removed from models. All post hoc analyses were adjusted for multiple comparisons using FDR (False Discovery Rate) corrections. Adjusted *P*‐values are denoted *P*ʹ.

### TEMPORAL ISOLATION

To assess whether *F. selysi* and *F. cinerea* colonies differed in the timing of production of winged females and males, we performed a permutation test in which the observed temporal overlap between species was compared to a null distribution. To obtain the null distribution, we first calculated the period in which winged females or males were observed for each colony. We then randomly allocated each colony to one or the other species and calculated the mean overlapping period between the two groups, repeating this process 10,000 times. We finally compared this null distribution to the observed overlap value.

### MATE CHOICE

We used Generalized Linear Mixed Models (GLMMs) with binomial error distribution to test if queens and males had mated assortatively with respect to their species (0 = disassortative mating, 1 = assortative mating). We excluded trials in which the queen did not mate. We fitted two models (one per queen's species) and included the colony of origin of the queens as a random factor. Because the population of origin and species were confounded in part of the trials (the queen and conspecific males often originated from the same population), we further examined whether queens and males had mated assortatively when considering only trials where the queen and conspecific males originated from the same population, and separately, only trials where they originated from different populations.

### GENETIC INCOMPATIBILITIES BETWEEN SPECIES

To detect potential genetic incompatibilities between species, we analyzed the reproductive success of queens using GLMMs. We included in this analysis *F. selysi* queens mated to either *F. selysi* (*n* = 134) or *F. cinerea* (*n* = 15) males, and *F. cinerea* queens mated to either *F. selysi* (*n* = 25) or *F. cinerea* (*n* = 24) males. Using a model with binomial error distribution, we assessed the probability that queens successfully produced brood, considering that queens failed when they died or did not produce an offspring before the end of the experiment. We then tested if the queens that successfully produced brood differed in the number of offspring they produced, using a model with Gaussian error distribution. The queen species, her mate species, and the interaction of these factors were included as fixed factors. The year of the experiment and the colonies of origin of the male and queen were included as random factors.

### DYADIC ENCOUNTERS

We compared the aggression indices (AIs) of workers with GLMMs. We used a Tweedie error distribution because our dataset comprised a mix of zeros and nonnegative continuous data points that could not be fitted to the normal distribution. We fitted one model per focal species. We included the species of the nonfocal worker as a fixed factor. To account for the nonindependence of observations, we included the colony of origin of the focal worker and the trial id as random factors. All assays in which workers interacted at least one time were included in these analyses.

### SPECIES RECOGNITION: GCMS ANALYSIS OF CHCs

To test for overall differences between the CHC profiles of the two pure species and their hybrid, we calculated Bray‐Curtis distances between chemical profiles (computed from relative abundances of peaks) and performed Permutational Multivariate Analysis of Variance (PERMANOVA) with the package “vegan” (Oksanen et al. 2019). We included the species of the sample as the explanatory variable (three levels: *F. selysi*, *F. cinerea*, and hybrid) and used 10,000 permutations. The two replicates of workers per colony were averaged prior to analysis to avoid pseudoreplication, which led to 15, 18, and 8 datapoints for *F. selysi*, *F. cinerea*, and hybrids, respectively. We also tested for differences between the two pure species and their hybrid in the relative proportion of each cuticular compound. We fitted one GLMM per compound and included the species of the sample as a fixed factor, and the colony of origin of the sample and the year it was extracted as random factors. We finally tested whether the Bray‐Curtis (chemical) distance between CHC profiles correlated with the Euclidean (genetic) distance between hybrid indices of colonies. To do this, we performed a Mantel test, using Spearman correlation and 100,000 permutations.

## Results

### TEMPORAL ISOLATION

We found no evidence that temporal isolation constitutes a premating barrier to hybridization. Overall, *F. selysi* and *F. cinerea* colonies did not differ significantly in their timing of production of females or males (Permutation test; *P* = 0.95; Fig. S1). Winged females or males were found inside 38.9% (14/36) and 56.3% (9/16) of the monitored colonies of *F. selysi* and *F. cinerea*, respectively.

### MATE CHOICE

**Figure 1:**
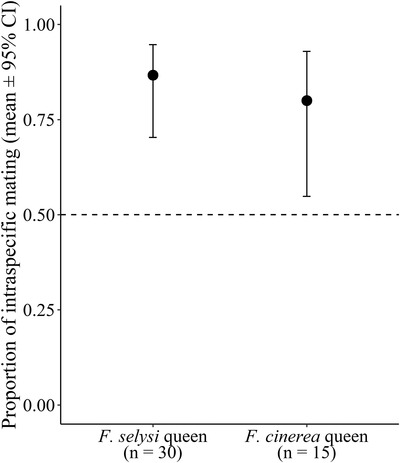
Mate choice of *F. selysi* and *F. cinerea* queens. The dashed line illustrates the expected proportion of intraspecific mating under random mating. *n* = number of successful mating trials.

Mating was mostly assortative (Fig. 1). In mate choice experiments, both *F. selysi* and *F. cinerea* queens were significantly more likely to mate with conspecific males than with heterospecific males (Estimate = 2.03, SE = 0.83, *z* = 2.45, *P* = 0.014 and Estimate = 1.39, SE = 0.65, *z* = 2.15, *P* = 0.032, respectively). *Formica selysi* queens mated with *F. selysi* males in 87% (26/30) of the mating events, whereas *F. cinerea* queens mated with *F. cinerea* males in 80% (12/15) of the mating events. Mating was assortative in trials where the queen and conspecific males originated from the same population (Estimate = 2.08, SE = 0.75, *z* = 2.77, *P* = 0.006) and in trials where the queens and conspecific males originated from different populations (Estimate = 1.61, SE = 0.77, *z* = 2.08, *P* = 0.038).

### GENETIC INCOMPATIBILITIES BETWEEN SPECIES

We did not detect postmating genetic incompatibilities between *F. selysi* and *F. cinerea*. Queens of each species did not differ significantly in their probability of surviving and producing brood (Estimate = 0.28, SE = 0.53, *z* = 0.52, *P* = 0.60; Fig. S2a), nor in the number of workers produced (Estimate = −0.48, SE = 1.02, *z* = −0.47, *P* = 0.64; Fig. S2b). The species of the queen's mate did not affect the queen's probability of producing brood (Estimate = −0.25, SE = 0.62, *z* = −0.41, *P* = 0.68; Fig. S2a) nor the number of offspring produced (Estimate = 0.79, SE = 0.83, *z* = 0.94, *P* = 0.35; Fig. S2b). More importantly, interspecific crosses did not show signs of genetic incompatibilities, as there was no significant interaction between the queen species and her mate species on the probability that the queens survived and produced brood (Estimate = 0.17, SE = 1.11, *z* = 0.15, *P* = 0.88) nor on the number of offspring produced by the queens (Estimate = −0.17, SE = 1.64, *z* = −0.10, *P* = 0.92). In addition, viable hybrid workers, winged queens, and males were repeatedly sampled in field colonies (Table S2).

### DYADIC ENCOUNTERS

**Figure 2:**
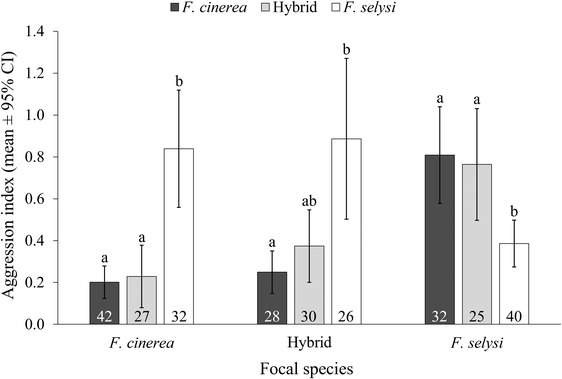
Aggression index of focal *F. cinerea*, hybrid, and *F. selysi* workers (*x*‐axis) according to the species of the opponent workers (*F. cinerea*: black bars; hybrids: light gray bars; *F. selysi*: white bars). Sample size is indicated within bars. Same letters within each focal species indicate lack of statistically significant differences (*P* > 0.05) after FDR correction for multiple comparisons.

In encounters with non‐nestmates, workers showed species recognition abilities (Fig. 2). Workers’ aggressivity varied according to the species of their opponents (focal species *F. cinerea*: *χ* ^2^ = 24.35, *P* < 0.0001; Hybrid: *χ* ^2^ = 9.2, *P* = 0.01; *F. selysi*: *χ* ^2^ = 9.95, *P* = 0.007; Fig. 2). Overall, *F. cinerea* workers showed little aggression toward conspecific workers and hybrid workers, but were aggressive toward *F. selysi* workers (post hoc analyses: *F. cinerea*‐*F. cinerea* vs. *F. cinerea*‐Hybrid: Estimate = 0.07, SE = 0.42, *z* = 0.15, *P* = 0.88; *F. cinerea*‐*F. selysi* vs. *F. cinerea*‐*F. cinerea*: Estimate = 1.47, SE = 0.36, *z* = 4.14, *P* < 0.001; *F. cinerea*‐*F. selysi* vs. *F. cinerea*‐Hybrid: Estimate = 1.54, SE = 0.39, *z* = 3.98, *P* < 0.001). By contrast, *F. selysi* workers were aggressive toward both *F. cinerea* and hybrid workers, but less aggressive toward conspecific workers (post hoc analyses: *F. selysi*‐*F. cinerea* vs. *F. selysi*‐Hybrid: Estimate = 0.12, SE = 0.24, *z* = 0.48, *P* = 0.63; *F. selysi*‐*F. selysi* vs. *F. selysi*‐*F. cinerea*: Estimate = −0.73, SE = 0.24, *z* = −3.05, *P* = 0.009; *F. selysi*‐*F. selysi* vs. *F. selysi*‐Hybrid: Estimate = −0.61, SE = 0.27, *z* = −2.32, *P* = 0.034). Reciprocally, hybrid workers were more aggressive toward *F. selysi* workers than toward *F. cinerea* workers, but were as aggressive toward other hybrid workers as toward workers of the two parent species (post hoc analyses: Hybrid‐*F. cinerea* vs. Hybrid‐Hybrid: Estimate = −0.32, SE = 0.42, *z* = −0.77, *P* = 0.44; Hybrid‐*F. selysi* vs. Hybrid‐*F. cinerea*: Estimate = 1.11, SE = 0.38, *z* = 2.92, *P* = 0.014; Hybrid‐*F. selysi* vs. Hybrid‐Hybrid: Estimate = 0.79, SE = 0.40, *z* = 1.97, *P* = 0.079). The level of aggression exhibited by each worker was positively correlated to the Bray‐Curtis distance between the hydrocarbon profile of its colony and that of its opponent (Mantel test: *r* = 0.36, *P* < 0.0001).

### CHCs PROFILES

**Figure 3:**
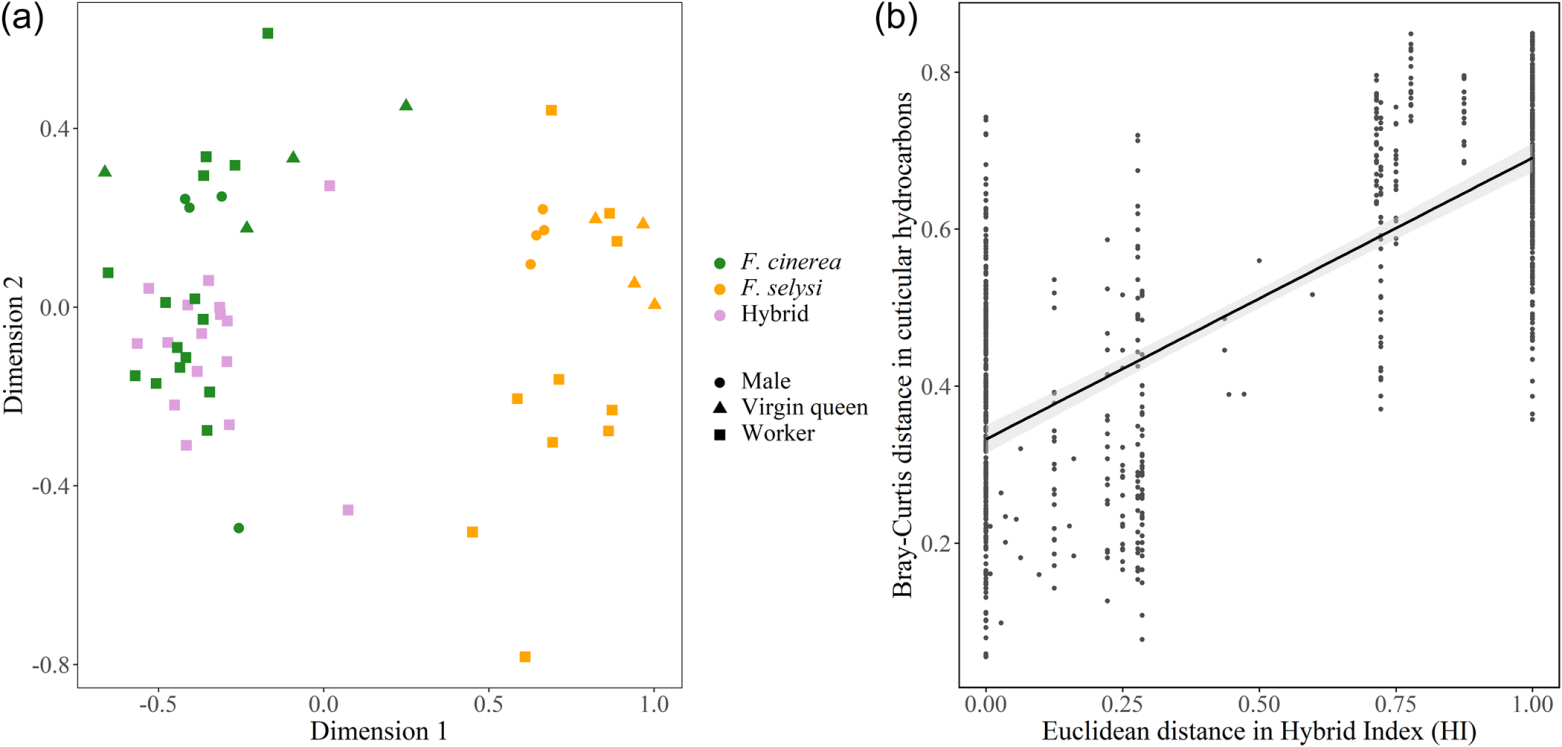
Proximity between cuticular hydrocarbon (CHCs) profiles of *F. cinerea*, *F. selysi*, and hybrid queens, males, and workers. Panel (a) shows Nonmetric Multidimensional Scaling (NMDS) plot of the Bray‐Curtis distance between CHCs. Each dot represents one winged queen (triangle), one virgin male (circle), or a pool of three workers (square) from *F. cinerea* (green, *n* = 23), *F. selysi* (yellow, *n* = 18), and their hybrid (purple, *n* = 16). *Formica selysi* individuals are perfectly discriminated from the other species and hybrids along the first dimensional axis, whereas *F. cinerea* and hybrids largely overlap. Panel (b) shows the positive correlation between the Bray‐Curtis distance in cuticular hydrocarbons of individuals and the Euclidean distance between the hybrid indices of their colonies. Each dot represents one pair of individuals.

The CHC profiles of individuals clustered according to species, but not according to sex or caste (Fig. 3a). Specifically, the CHC profiles differed between species, and between *F. selysi* and hybrids, but they did not differ between *F. cinerea* and hybrids (*F*_2, 40_ = 21.05, *P* < 0.0001; post hoc comparisons: *P*ʹ = 0.003, *P*ʹ = 0.003, and *P*ʹ = 0.45, respectively; Figs. 3a, S3, S4). This pattern is consistent with the hypothesis that CHCs play a role in assortative mate choice of queens and males, as well as in aggression between workers in dyadic encounters. Chemical differences between species were both qualitative and quantitative, with 42 compounds out of 74 (56.8%) that were either exclusive to one species or were present in the two species in statistically significantly different relative proportions (Table S3). The chemical distance between samples was positively correlated to the distance between the hybrid indices of their colonies (Mantel test: *r* = 0.71, *P* < 0.0001; Fig. 3b). This positive correlation was also present when considering hybrids only (*n* = 8 colonies, *r* = 0.70, *P* = 0.012; Fig. S5).

### Discussion

A mosaic hybrid zone characterized by a low level of asymmetric hybridization between two ant species provides a rare opportunity to study the evolutionary mechanisms maintaining the genetic integrity of hybridizing species (Purcell et al. 2016; Irwin 2020). We studied potential pre‐ and postmating barriers to hybridization between these two species, *F. selysi* and *F. cinerea*. Both species produced queens or males simultaneously, which suggests that temporal isolation does not prevent interspecific mating in the field. In mate choice experiments, mating was strongly assortative. Queens and males of each species preferentially mated with conspecifics, which likely plays a role in keeping hybridization low and preventing genetic admixture. We found no evidence for genetic incompatibility reducing the fertility of queens mated to heterospecific males: their hybrid offspring workers were as numerous as pure‐bred offspring workers. CHCs differed markedly between species, both in composition and relative abundances, and independently of the individuals’ caste and sex. This pattern suggests that species‐specific hydrocarbon cues may serve for species recognition and assortative mate choice. In line with this hypothesis, workers’ behavior also correlated with species‐specific CHCs. Workers discriminated *F. selysi* from both *F. cinerea* and hybrid workers, which had dissimilar CHC profiles, but they did not discriminate *F. cinerea* from hybrid workers, which had similar CHC profiles. This asymmetric recognition between hybrids and members of the parent species is consistent with the documented asymmetric hybridization pattern (Purcell et al. 2016). We propose that the CHC‐based recognition system of ants facilitated species recognition and the establishment of assortative mate preference.

In many insects, temporal isolation is a key barrier to interspecific mating (Harrison and Arnold 1982; Harrison 1985; Hölldobler and Wilson 1990; Ramsey et al. 2003). The monitoring of queen and male production by field colonies showed that *F. selysi* and *F. cinerea* reproductive individuals are produced in synchrony. Although subtle differences in timing might still contribute to restrict hybridization, the fact that the nuptial flights of both species occur during the same period suggests that temporal isolation does not constitute a strong barrier to hybridization between these species.

With mate choice experiments, we found that queens and males of *F. selysi* and *F. cinerea* preferentially mated with conspecifics. On average, 84% of all matings were intraspecific, whereas there were equal opportunities for interspecific mating. Strong preference for conspecifics is in accordance with the relative rarity of hybrids in the wild (Purcell et al. 2016). In ants, females typically attract males using volatile sex pheromones (e.g., Walter et al. 1993). At closer range, nonvolatile chemical cues, in particular CHCs, may serve for mate recognition (reviewed in Ayasse et al. 2001; Howard and Blomquist 2005; Weiss et al. 2015). We found that *F. selysi* and *F. cinerea* queens, males, and workers carry species‐specific CHC profiles, which confirms and expands to queens and males previous evidence based on workers only (Purcell et al. 2016). These CHCs convey information about species membership and may be the recognition cues underlying assortative mate choice by *F. selysi* and *F. cinerea*.

Theory and empirical data suggest that assortative mate choice co‐evolves with genetic incompatibilities, in a reinforcing feedback loop (Liou and Price 1994; Servedio and Noor 2003; Albert and Schluter 2004; Shizuka and Hudson 2020). In short, hybridization costs select for intraspecific mate choice, which in turn limits gene flow and increases genetic differentiation between sister species, further enlarging the costs of hybridization and facilitating species recognition (Coyne et al. 2004). We did not detect any significant hybridization cost when comparing the fertility of interspecific crosses to that of intraspecific crosses. Mating with the other species did not decrease the queens’ likelihood of producing brood nor the number of adult workers produced. Moreover, genetic analyses of individuals collected in the field revealed that F1 and backcrossed hybrid workers, males, and winged females are viable in nature (Purcell et al. 2016; this study). In the few experimental studies of interspecific mating that have been conducted in ants, the outcomes were highly variable, ranging from complete lethality to fully viable hybrids (Feldhaar et al. 2008). Although we did not detect hybridization costs in our breeding experiments, deleterious effects might appear with backcrosses (Schwander et al. 2008), or when producing queens and males rather than workers (Kulmuni and Pamilo 2014). Hybridization costs are also likely to be higher in other social and ecological conditions, for example, during independent colony founding by queens in harsh field conditions.

Moderate hybridization costs might suffice to select for divergent CHC profiles. Alternatively, hydrocarbon cues may diverge between lineages as a result of genetic drift, local adaption, or sexual selection, independently of hybridization costs (Gleason et al. 2009; Drescher et al. 2010; Schwander et al. 2013; Dembeck et al. 2015). Increasing divergence in CHC profiles can result in assortative mating, which in turn increases reproductive isolation (Blows and Allan 1998; Schwander et al. 2013; Maroja et al. 2014). The efficient CHC‐based recognition system of ants may thus lead queens and males to preferentially mate with conspecifics even when hybridization costs are minimal.

The behavioral interactions between hybrid workers and workers of each parent species were asymmetric. Hybrid and *F. cinerea* workers usually interacted peacefully but responded aggressively to *F. selysi* workers. This behavioral pattern matches the CHC profiles, with hybrid workers being more like *F. cinerea* than *F. selysi*. It is also in line with their genetic background, as the large majority of hybrids were genetically closer to *F. cinerea* than to *F. selysi*, in accordance with previous observations (Purcell et al. 2016). The factors causing this skewed distribution are yet unknown. The *F. cinerea* CHC profile might be dominant in F1 hybrids, which would favor subsequent backcrosses with *F. cinerea*. The high correlation between the CHC profile of individuals and the hybrid index of their colonies provides no support to this hypothesis, and rather suggests that CHCs of *F. cinerea* and *F. selysi* are co‐dominant in hybrids. It also confirms that CHCs are to some extent genetically determined (van Zweden et al. 2009; Martin et al. 2013; Holze et al. 2021). Whatever the mechanism and causal relationships, the chemical and genetic proximity between hybrids and *F. cinerea* is associated with an asymmetric response of hybrid workers toward their parent species, and potentially a biased mate choice of hybrid males and queens toward *F. cinerea*. This likely impacts the dynamics of the hybrid zone by reinforcing the introgression of *F. selysi* alleles into *F. cinerea*, which might in turn lead to asymmetric hybridization costs between these species.

In conclusion, we uncovered strong, although incomplete, assortative mate choice in two hybridizing ant species, *F. selysi* and *F. cinerea*. The marked preference to mate with conspecifics helps explain the low frequency of hybrids in nature (Purcell et al. 2016). The fact that no genetic incompatibilities between species were detected suggests that assortative mate choice evolved in the absence of reinforcement (Servedio and Noor 2003). We propose that the efficient CHC‐based recognition system of ants can lead to assortative mate preference even when costs of hybridization are low. Asymmetries in CHC profiles and aggression between hybrid, *F. cinerea*, and *F. selysi* workers are also in line with asymmetric hybridization, skewed toward *F. cinerea*. These two ant species appear to have effective recognition systems that affect both worker behavior and mate choice, with consequences at the group, population, and species levels.

## AUTHOR CONTRIBUTIONS

PB, SZ, JP, AB, and MC planned and designed the study. PB, SZ, and TOH performed field sampling and laboratory work with the help of AA, JP, and AB. SZ and AA performed the mating experiment and the hybrid viability experiment. PB and TOH performed and analyzed the aggression tests. PB and SZ performed CHC extraction. GBR, DS, and PB performed the CHC analysis. PB and SZ analyzed the data. PB, SZ, and MC wrote the manuscript, with contribution of all the authors. All authors read and approved the manuscript.

## CONFLICT OF INTEREST

The authors declare no conflict of interest.

## DATA ARCHIVING

Analyses reported in this article can be reproduced using the data provided by Blacher et al. (2022) (https://doi.org/10.5061/dryad.s1rn8pkbn).

Associate Editor: S. Foitzik

Handling Editor: T. Chapman

## Supporting information

Supplementary Information
