# Supplementary material for: Species recognition limits mating between hybridizing ant species
Source: Evolution. 2022 Jul 19;76(9):2105–15. doi: 10.1111/evo.14566 (PMC9541793; doi:10.1111/evo.14566)
Supplement: Supplementary file 1 — Supplementary Information [file EVO-76-2105-s001.pdf]

Supplementary material

Title: Species recognition limits mating between hybridizing ant species

Running title: Species recognition in hybridizing ant species

Pierre Blacher[*1], Sacha Zahnd*[1], Jessica Purcell[2], Amaury Avril[1], Thalita Oliveira Honorato[1], Gaëlle Bailat-Rosset[3], Davide Staedler[3,4], Alan Brelsford[5] and Michel Chapuisat[1]

* should be considered joint first author

Authors' affiliations

[1]Departement of Ecology and Evolution, University of Lausanne, 1015 Lausanne, Switzerland

[2]Department of Entomology, University of California, Riverside, Riverside, CA 92521, USA

[3]Scitec Research SA, Av. De Provence 18, 1007 Lausanne, Switzerland

[4]Department of Biomedical Sciences, University of Lausanne, 1011 Lausanne, Switzerland

[5]Department of Biology, University of California, Riverside, Riverside, CA 92521, USA

Corresponding authors

pierre.blacher@unil.ch, sacha.zahnd@unil.ch and michel.chapuisat@unil.ch

Tables

Table S1. Number of queens included in the genetic incompatibility experiment

	F. selysi queens		*F. cinerea* queens	
Mated with	*F. selysi*	*F. cinerea*	*F. selysi*	*F. cinerea*
Total	134	15	25	24
Alive after 6 weeks	126	15	21	24
Producing brood	68	8	12	11

Table S2. Field monitoring of hybrid colonies and production of hybrid winged queens and males

Year	Population	No. of field visits	No. of colonies visited	No. of colonies producing sexuals	No. of hybrid winged queens	No. of hybrid males
2014	Riddes	10	41	4	13	19
2014	Saillon	10	40	1	4	0
2015	Branson	13	27	8	6	9
2018	Branson	6	22	9	23	16

Table S3. Relative abundance of peaks (percentage of total area) in extracts of cuticular hydrocarbons (CHCs) of workers, virgin queens and males from *F. selysi*, *F. cinerea a*nd hybrids. Peaks in bold present statistical differences between species: ¥ symbol indicates statistical difference between *F. selysi* and both *F. cinerea* and hybrids and § symbol indicates difference between *F. selysi* and *F. cinerea* only. None of the peaks present statistical difference between *F. cinerea* and hybrids.

No.	Name	RT	*Formica cinerea*			Hybrid	*Formica selysi*			
			Worker (*n=15*)	Queen (*n=4*)	Male (*n=4*)	Worker (*n=16*)	Worker (*n=10*)	Queen (*n=4*)	Male (*n=4*)	
P1	Nonanal	5.77	0.37 ± 0.1	0.1 ± 0.02	0.22 ± 0.13	0.38 ± 0.13	2.05 ± 1.42	0.16 ± 0.04	0.04 ± 0.01	
P2	*n*-C$_{12}$	8.35	0.1 ± 0.02	0.06 ± 0.01	0.04 ± 0.01	0.24 ± 0.06	0.11 ± 0.04	0.06 ± 0.02	0.08 ± 0.02	
P3	*n*-C$_{14}$	11.43	0.21 ± 0.04	0.09 ± 0.01	0.06 ± 0.02	0.28 ± 0.04	0.22 ± 0.05	0.09 ± 0.02	0.11 ± 0.02	
P4	*n*-C$_{15}$	12.44	0.21 ± 0.04	0.17 ± 0.04	0.14 ± 0	0.27 ± 0.05	0.21 ± 0.04	0.18 ± 0.06	0.26 ± 0.05	
P5	*n*-C$_{17}$	16.84	0.31 ± 0.06	0.09 ± 0.02	0.15 ± 0.09	0.39 ± 0.06	0.22 ± 0.07	0.07 ± 0.02	0.09 ± 0.02	
P6	2-meC$_{17}$	18.07	0.35 ± 0.06	0.15 ± 0.05	0.33 ± 0.22	0.4 ± 0.06	0.24 ± 0.06	0.15 ± 0.05	0.14 ± 0.02	
P7	x-C$_{18:1}$	19.21	0.17 ± 0.03	0.04 ± 0.02	0.08 ± 0.05	0.2 ± 0.02	0.14 ± 0.04	0.04 ± 0.02	0.05 ± 0.01	
P8	*n*-C$_{18}$	19.65	0.14 ± 0.06	0 ± 0	0 ± 0	0.17 ± 0.05	0.09 ± 0.05	0 ± 0	0 ± 0	
P9	Hexadecanal	19.90	0.15 ± 0.02	0.03 ± 0.01	0.02 ± 0.01	0.25 ± 0.03	0.11 ± 0.02	0.03 ± 0.02	0.04 ± 0	
P10	unknown	22.89	0.47 ± 0.08	0.02 ± 0.02	0.08 ± 0.06	0.54 ± 0.08	0.28 ± 0.1	0.03 ± 0.02	0.05 ± 0.01	
P11	*n*-C$_{19}$	23.06	0.24 ± 0.05	0.04 ± 0.01	0.1 ± 0.07	0.29 ± 0.04	0.16 ± 0.04	0.04 ± 0.01	0.04 ± 0.01	
P12	x-meC$_{19}$	24.32	0.38 ± 0.05	0.17 ± 0.07	0.53 ± 0.39	0.47 ± 0.06	0.39 ± 0.12	0.14 ± 0.04	0.25 ± 0.05	
**P13**	**Octadecanal**	**25.94**	**0.06 ± 0.02**	**0 ± 0**	**0 ± 0**	**0.2 ± 0.08**	**0.51 ± 0.14**	**0.23 ± 0.14**	**0.15 ± 0.01**	**¥**
**P14**	***n*-C$_{21}$**	**28.25**	**0.23 ± 0.03**	**0.43 ± 0.08**	**0.92 ± 0.07**	**0.34 ± 0.17**	**0.02 ± 0.01**	**0.08 ± 0.03**	**0.38 ± 0.05**	**¥**
P15	Octadecenoic acid	29.37	0.19 ± 0.08	0.06 ± 0.03	0.29 ± 0.21	0.33 ± 0.1	0.47 ± 0.21	0.07 ± 0.03	0.09 ± 0.02	
**P16**	**Unknown + 2-propyl hexadecanoate**	**29.49**	**1.7 ± 0.35**	**0.9 ± 0.52**	**1.19 ± 0.35**	**1.47 ± 0.32**	**0.19 ± 0.09**	**0.08 ± 0.05**	**0.13 ± 0.02**	**¥**
P17	3-meC$_{21}$	30.46	0.16 ± 0.02	0.09 ± 0.05	0.22 ± 0.14	0.17 ± 0.03	0.11 ± 0.02	0.08 ± 0.04	0.14 ± 0.03	
**P18**	***n*-C$_{22}$**	**31.10**	**0.44 ± 0.04**	**0.48 ± 0.05**	**0.57 ± 0.05**	**0.91 ± 0.52**	**0.09 ± 0.02**	**0.08 ± 0.03**	**0.18 ± 0.02**	**§**
**P19**	**x-meC$_{22}$**	**32.19**	**0.44 ± 0.06**	**0.22 ± 0.12**	**0.36 ± 0.07**	**0.5 ± 0.11**	**0.04 ± 0.03**	**0 ± 0**	**0.02 ± 0.01**	**¥**
**P20**	**x'-meC$_{22}$**	**32.38**	**1.69 ± 0.21**	**1.4 ± 0.57**	**1.29 ± 0.23**	**1.32 ± 0.2**	**0.01 ± 0.01**	**0.01 ± 0.01**	**0 ± 0**	**¥**
**P21**	**unknown**	**32.74**	**0.14 ± 0.02**	**0.48 ± 0.18**	**0.27 ± 0.09**	**0.23 ± 0.05**	**0.02 ± 0.01**	**0 ± 0**	**0 ± 0**	**§**
P22	9-C$_{23:1}$	33.07	2.16 ± 0.6	1.09 ± 0.2	0.89 ± 0.15	1.66 ± 0.27	1.09 ± 0.22	1.68 ± 0.28	1.82 ± 0.15	
P23	7-C$_{23:1}$	33.27	0.31 ± 0.04	0.29 ± 0.08	0.31 ± 0.03	1.72 ± 1.47	0.01 ± 0	0.05 ± 0.02	0.15 ± 0.09	
**P24**	***n*-C$_{23}$**	**33.93**	**15.32 ± 1.12**	**15.44 ± 1.77**	**14.67 ± 1.76**	**12.25 ± 1.14**	**2.56 ± 0.53**	**2.99 ± 0.15**	**5.83 ± 0.48**	**¥**
P25	7-meC$_{23}$	34.35	0.65 ± 0.05	0.35 ± 0.2	1.32 ± 1.07	0.56 ± 0.04	0.35 ± 0.07	0.31 ± 0.22	0.71 ± 0.1	
**P26**	**5-meC$_{23}$**	**34.84**	**0.36 ± 0.06**	**0.9 ± 0.11**	**0.58 ± 0.12**	**0.4 ± 0.08**	**0.17 ± 0.09**	**0.49 ± 0.3**	**0.51 ± 0.07**	**¥**
**P27**	**2-meC$_{23}$ + unknown**	**35.06**	**14.45 ± 1.45**	**13.59 ± 4.79**	**14.17 ± 1.4**	**16.53 ± 1.81**	**1.42 ± 0.93**	**0.2 ± 0.13**	**0.08 ± 0.01**	**¥**
**P28**	**3-meC$_{23}$**	**35.81**	**1.36 ± 0.13**	**2.42 ± 0.65**	**1.62 ± 0.25**	**1.32 ± 0.1**	**0.18 ± 0.03**	**0.26 ± 0.05**	**0.3 ± 0.01**	**¥**
**P29**	**unknown**	**35.96**	**0.99 ± 0.19**	**1.35 ± 0.48**	**0.4 ± 0.06**	**0.54 ± 0.11**	**0 ± 0**	**0 ± 0**	**0 ± 0**	**§**
**P30**	**5,13-dimeC$_{23}$**	**36.09**	**0.14 ± 0.02**	**0.35 ± 0.11**	**0.33 ± 0.07**	**0.23 ± 0.06**	**0.01 ± 0.01**	**0.04 ± 0.03**	**0.08 ± 0.01**	**§**
**P31**	***n*-C$_{24}$ + Dioctyl adipate**	**36.55**	**7.27 ± 1.89**	**3.37 ± 0.44**	**2.59 ± 0.26**	**4.27 ± 1.14**	**4.19 ± 1.59**	**0.51 ± 0.06**	**0.96 ± 0.04**	**§**
P32	x-meC$_{24}$	36.76	3.23 ± 3.02	0.41 ± 0.02	0.12 ± 0.06	4.25 ± 2.73	0.04 ± 0.02	0.02 ± 0.02	0.05 ± 0.02	
**P33**	**x'-meC$_{24}$**	**37.55**	**1.1 ± 0.12**	**0.45 ± 0.36**	**0.39 ± 0.13**	**1.08 ± 0.09**	**0 ± 0**	**0 ± 0**	**0 ± 0**	**¥**
**P34**	**x''-meC$_{24}$**	**37.72**	**2.38 ± 0.24**	**1.82 ± 0.59**	**2.39 ± 0.17**	**2.38 ± 0.18**	**0.02 ± 0.02**	**0.03 ± 0.02**	**0 ± 0**	**¥**
**P35**	**unknown**	**38.03**	**0.32 ± 0.08**	**1.04 ± 0.25**	**0.47 ± 0.14**	**0.58 ± 0.14**	**0.17 ± 0.07**	**0.08 ± 0.02**	**0.09 ± 0.01**	**§**
**P36**	**9-C$_{25:1}$**	**38.41**	**1.06 ± 0.14**	**1.61 ± 0.48**	**0.75 ± 0.11**	**3.08 ± 0.94**	**4.89 ± 0.84**	**6.96 ± 2.86**	**8.39 ± 0.58**	**§**
**P37**	**7-C$_{25:1}$**	**38.58**	**1.6 ± 0.17**	**1.29 ± 0.29**	**1.32 ± 0.12**	**1.22 ± 0.16**	**0.47 ± 0.31**	**2.42 ± 2.17**	**0.26 ± 0.03**	**§**
P38	*n*-C$_{25}$	39.14	11.52 ± 1.69	16.75 ± 4.33	13.59 ± 0.17	11.56 ± 0.96	15.09 ± 3.38	10.12 ± 2.72	22.42 ± 0.79	
**P39**	**13-,11-,9-meC$_{25}$**	**39.98**	**1.27 ± 0.09**	**1.66 ± 0.23**	**1.33 ± 0.29**	**1.28 ± 0.09**	**0.34 ± 0.11**	**1 ± 0.29**	**1.44 ± 0.12**	**¥**
**P40**	**7-meC$_{25}$**	**40.18**	**2.11 ± 0.23**	**1.91 ± 0.31**	**2.18 ± 0.46**	**3 ± 0.39**	**0.1 ± 0.06**	**0.1 ± 0.02**	**0.12 ± 0.02**	**¥**
P41	5-meC$_{25}$	40.41	0.06 ± 0.02	0.42 ± 0.09	0.16 ± 0.06	0.28 ± 0.07	0.03 ± 0.02	0.12 ± 0.05	0.16 ± 0.02	
**P42**	**3-meC$_{25}$**	**41.08**	**12.3 ± 1.31**	**10.53 ± 2.71**	**11.72 ± 0.87**	**13.49 ± 0.83**	**0.58 ± 0.18**	**1.59 ± 0.37**	**1.74 ± 0.07**	**¥**
**P43**	**unknown**	**41.29**	**1.8 ± 1.33**	**0.87 ± 0.11**	**0.59 ± 0.04**	**0.65 ± 0.07**	**0.06 ± 0.02**	**0.21 ± 0.04**	**0.25 ± 0.02**	**§**
P44	*n*-C$_{26}$	41.68	0.48 ± 0.07	1.15 ± 0.33	0.79 ± 0.21	0.49 ± 0.04	0.63 ± 0.17	0.31 ± 0.1	0.86 ± 0.08	

P45	unknown	41.86	0.01 ± 0.01	0.41 ± 0.28	0 ± 0	0.17 ± 0.08	0.12 ± 0.04	0.37 ± 0.1	0.59 ± 0.09	
**P46**	**x,y-$C_{27:2}$**	**43.07**	**0.01 ± 0.01**	**0 ± 0**	**0 ± 0**	**0.01 ± 0.01**	**0.43 ± 0.11**	**1.23 ± 0.44**	**0.95 ± 0.11**	**¥**
P47	x',y'-$C_{27:2}$	43.54	1.43 ± 0.55	0.19 ± 0.08	0.11 ± 0.06	0.51 ± 0.12	0.26 ± 0.04	0.68 ± 0.24	0.8 ± 0.06	
P48	9,7-$C_{27:1}$	43.79	0.52 ± 0.05	0.17 ± 0.06	0.3 ± 0.08	0.6 ± 0.21	11.36 ± 4.86	6.58 ± 6.58	0 ± 0	
**P49**	**5-$C_{27:1}$**	**44.04**	**0.21 ± 0.09**	**0.06 ± 0.05**	**0 ± 0**	**0.01 ± 0.01**	**10.09 ± 5.1**	**10.1 ± 9.28**	**0.86 ± 0.03**	**§**
**P50**	***n*-C_{27}**	**44.36**	**2.97 ± 0.63**	**7.06 ± 2.16**	**10.06 ± 3.38**	**2.8 ± 0.32**	**11.88 ± 2.93**	**7.28 ± 2.28**	**12.47 ± 0.66**	**§**
**P51**	**x,y-$C_{27:2}$**	**44.75**	**0.02 ± 0.02**	**0 ± 0**	**0 ± 0**	**0 ± 0**	**0.31 ± 0.03**	**0.58 ± 0.14**	**0.53 ± 0.03**	**¥**
P52	unknown	45.00	0.39 ± 0.18	0.14 ± 0.13	0.05 ± 0.04	0.36 ± 0.12	0.05 ± 0.04	0 ± 0	0.04 ± 0.03	
**P53**	**11-meC_{27}**	**45.17**	**0.18 ± 0.12**	**0.38 ± 0.2**	**0.21 ± 0.02**	**0.21 ± 0.08**	**0.5 ± 0.24**	**1.91 ± 0.43**	**1.53 ± 0.1**	**§**
P54	9-meC_{27}	45.51	0.89 ± 0.41	0.4 ± 0.12	0.9 ± 0.3	0.28 ± 0.09	0.32 ± 0.1	0.5 ± 0.11	0.48 ± 0.05	
P55	7-meC_{27}	45.80	0.21 ± 0.09	0.19 ± 0.11	0.11 ± 0.02	0.19 ± 0.05	0.25 ± 0.05	0.44 ± 0.08	0.49 ± 0.03	
P56	3-meC_{27}	46.35	0.41 ± 0.08	1.34 ± 0.33	0.32 ± 0.04	0.41 ± 0.11	0.7 ± 0.13	0.87 ± 0.15	0.79 ± 0.08	
**P57**	**2-meC_{27}**	**46.68**	**0.02 ± 0.01**	**0.14 ± 0.08**	**0.04 ± 0.03**	**0.03 ± 0.02**	**0.58 ± 0.11**	**0.98 ± 0.21**	**0.71 ± 0.05**	**¥**
**P58**	**x,y-dimeC_{28}**	**47.08**	**0.27 ± 0.05**	**0.4 ± 0.14**	**0.49 ± 0.16**	**0.21 ± 0.03**	**0.06 ± 0.03**	**0.06 ± 0.05**	**0.25 ± 0.03**	**§**
**P59**	**x',y'-dimeC_{28}**	**47.27**	**0.06 ± 0.03**	**0.15 ± 0.13**	**0 ± 0**	**0.03 ± 0.02**	**0.49 ± 0.09**	**0.9 ± 0.2**	**0.83 ± 0.09**	**¥**
P60	unknown	47.74	0.31 ± 0.11	0 ± 0	0 ± 0	0.16 ± 0.06	0.02 ± 0.02	0 ± 0	0 ± 0	
**P61**	**9,19-$C_{29:2}$**	**48.44**	**0.01 ± 0.01**	**0 ± 0**	**0 ± 0**	**0.03 ± 0.02**	**1.52 ± 0.47**	**3.24 ± 1.02**	**2.46 ± 0.14**	**¥**
**P62**	**9,21-$C_{29:2}$**	**48.76**	**0 ± 0**	**0.06 ± 0.03**	**0.02 ± 0.01**	**0 ± 0**	**3.18 ± 0.73**	**6.4 ± 2.42**	**5.01 ± 0.2**	**¥**
**P63**	**9,23-$C_{29:2}$**	**48.94**	**0.47 ± 0.21**	**0.21 ± 0.11**	**0.33 ± 0.24**	**0.1 ± 0.04**	**3.61 ± 0.64**	**6.06 ± 2.1**	**4.89 ± 0.15**	**¥**
**P64**	**7-$C_{29:1}$**	**49.32**	**0.01 ± 0.01**	**0.89 ± 0.61**	**0.15 ± 0.09**	**0.25 ± 0.17**	**7.21 ± 2.12**	**9.63 ± 3.26**	**8.48 ± 0.58**	**¥**
**P65**	**5-$C_{29:1}$**	**49.63**	**0 ± 0**	**0 ± 0**	**0 ± 0**	**0.01 ± 0.01**	**0.7 ± 0.13**	**0.81 ± 0.34**	**0 ± 0**	**¥**
P66	*n*-C_{29}	49.95	0.29 ± 0.11	2.16 ± 0.97	3.57 ± 1.3	0.39 ± 0.07	1.4 ± 0.3	1.57 ± 0.37	3.25 ± 0.4	
**P67**	**13-meC_{29}**	**50.83**	**0 ± 0**	**0.28 ± 0.19**	**0 ± 0**	**0 ± 0**	**0.47 ± 0.1**	**1.04 ± 0.07**	**0.48 ± 0.04**	**¥**
**P68**	**11,9-meC_{29}**	**52.93**	**0.03 ± 0.02**	**0.2 ± 0.09**	**0 ± 0**	**0.02 ± 0.02**	**0.28 ± 0.07**	**0.34 ± 0.07**	**0.41 ± 0.06**	**¥**
**P69**	**9,19-$C_{31:2}$**	**54.19**	**0 ± 0**	**0 ± 0**	**0 ± 0**	**0 ± 0**	**1.05 ± 0.25**	**1.06 ± 0.32**	**0.69 ± 0.04**	**¥**
**P70**	**9,21-$C_{31:2}$**	**54.40**	**0 ± 0**	**0 ± 0**	**0 ± 0**	**0 ± 0**	**2.51 ± 0.69**	**3.48 ± 0.82**	**3.01 ± 0.26**	**¥**
**P71**	**9-$C_{31:1}$**	**54.89**	**0 ± 0**	**0.48 ± 0.48**	**0.21 ± 0.13**	**0.04 ± 0.03**	**0.75 ± 0.22**	**1.63 ± 0.46**	**0.57 ± 0.15**	**¥**
P72	Cholesterol	55.10	0.68 ± 0.15	0.38 ± 0.24	1.79 ± 0.25	0.7 ± 0.06	0.51 ± 0.17	0.24 ± 0.2	0.32 ± 0.04	
P73	Cholesteryl propionate	56.92	0.21 ± 0.05	0.28 ± 0.15	1.8 ± 0.57	0.32 ± 0.08	0.42 ± 0.14	0.24 ± 0.07	0.33 ± 0.12	
**P74**	**9,21-$C_{33:2}$**	**59.47**	**0 ± 0**	**0 ± 0**	**0 ± 0**	**0 ± 0**	**0.92 ± 0.3**	**0.65 ± 0.26**	**0.33 ± 0.04**	**¥**

Figures

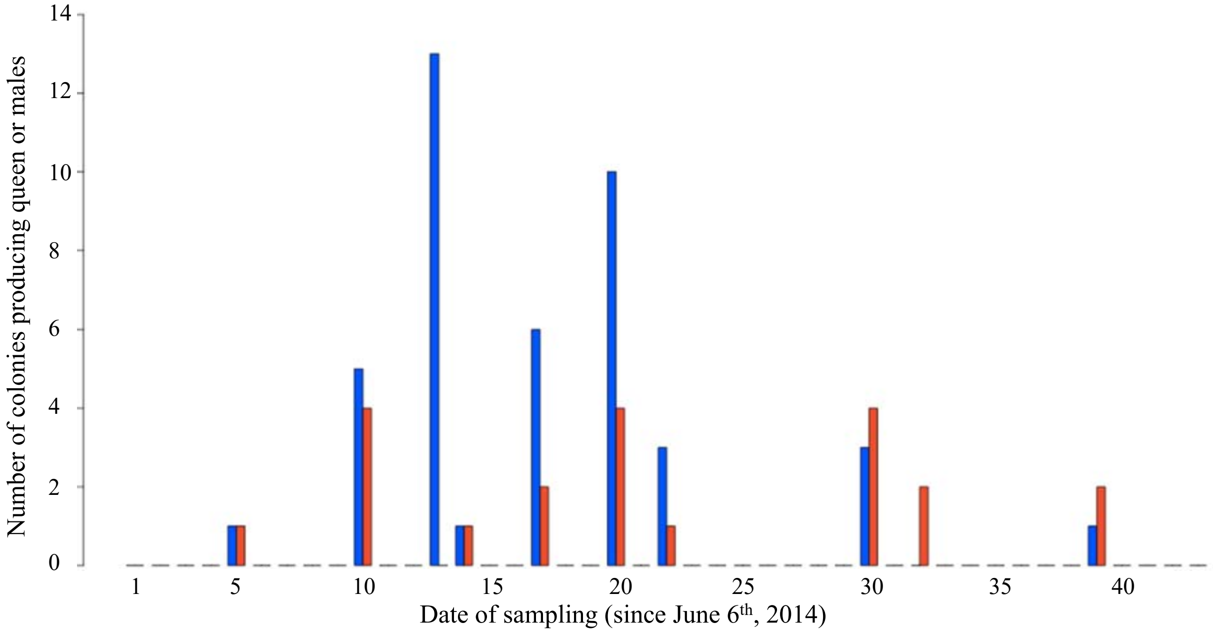

Figure S1. Timing of production of queens or males by *F. selysi* (blue) and *F. cinerea* (red) colonies. The number of colonies producing queens or males is plotted against the date of sampling (number of days since June 6th, 2014).

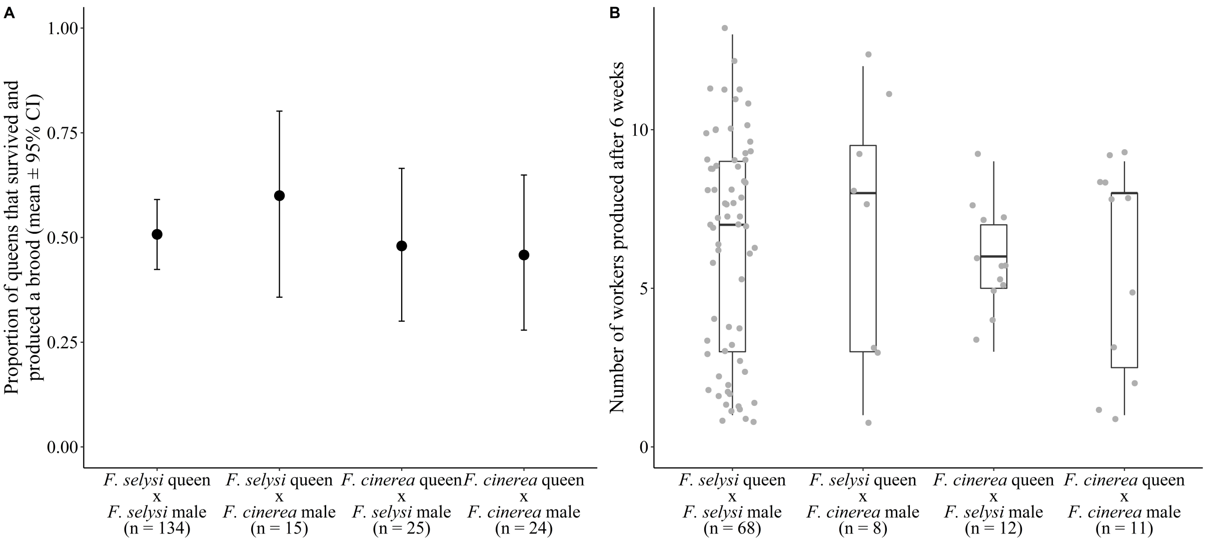

Figure S2. Reproductive success of queens according to their species and the species of the male with whom they mated. Plot (A) shows the success of the queens at surviving and producing brood after 6 weeks. Data are shown as mean ± 95% CI. Plot (B) shows the number of workers produced after 6 weeks by the queens that survived and produced brood. Horizontal lines represent the median and the boxes represent the 1st and 3rd quartiles.

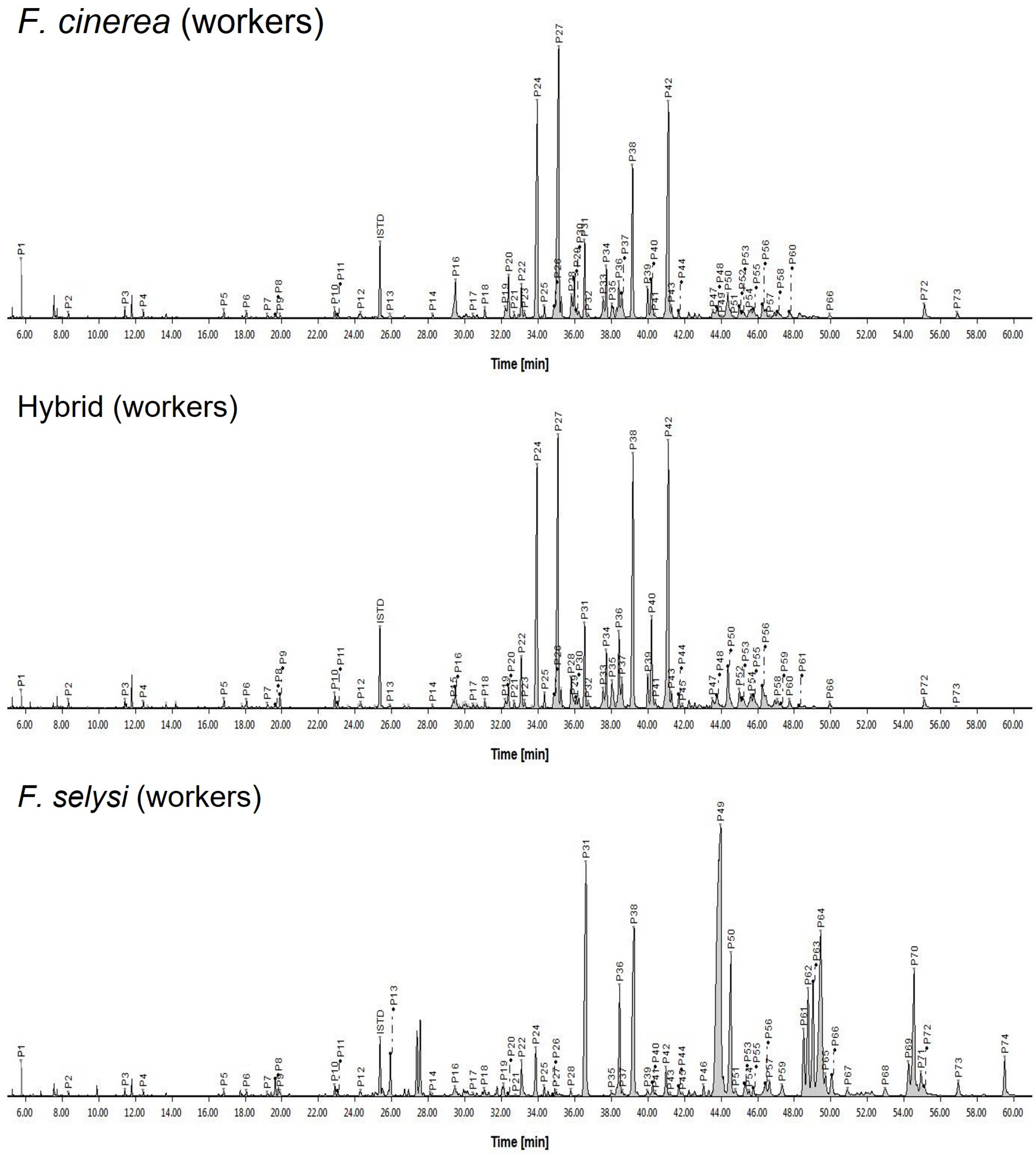

Figure S3. Example chromatograms of *F. cinerea*, hybrid and *F. selysi* workers (collected in 2017). Peak names with reference numbers are given in table S3. ISTD = internal standard (*n*-C20).

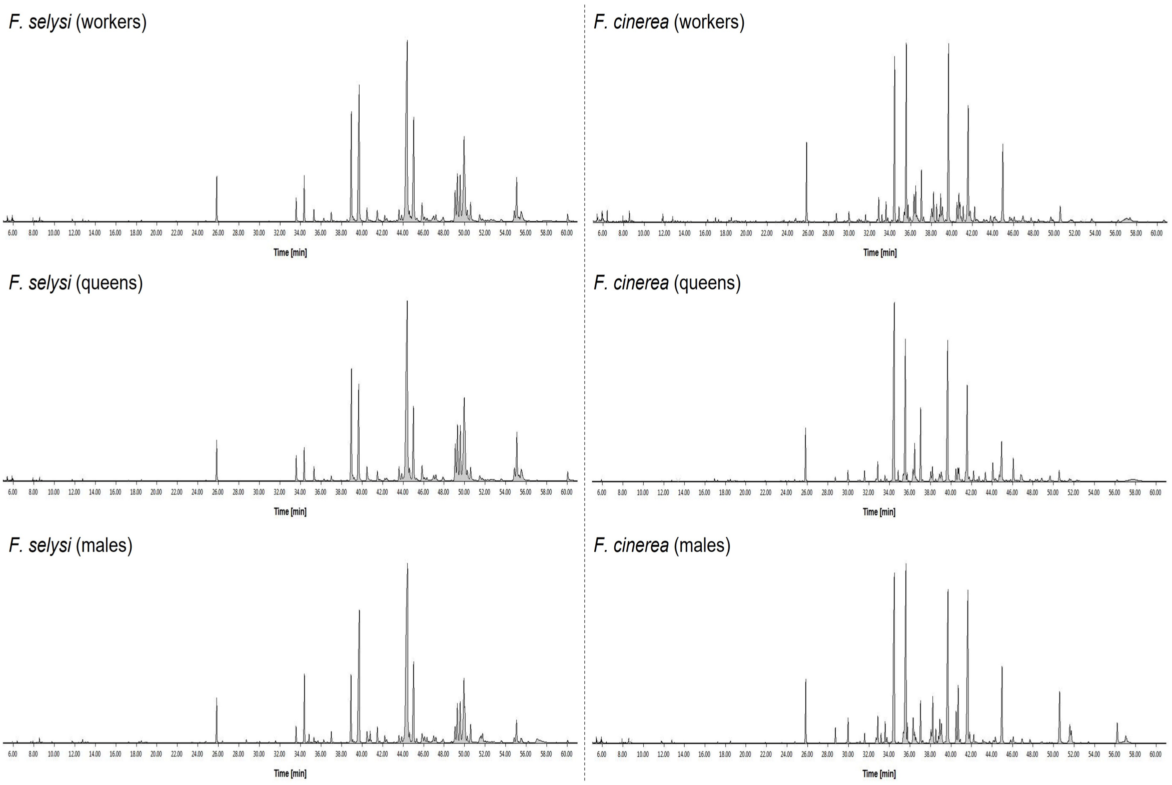

Figure S4. Example chromatograms of *F. selysi* and *F. cinerea* workers, virgin queens and virgin males (collected in 2021).

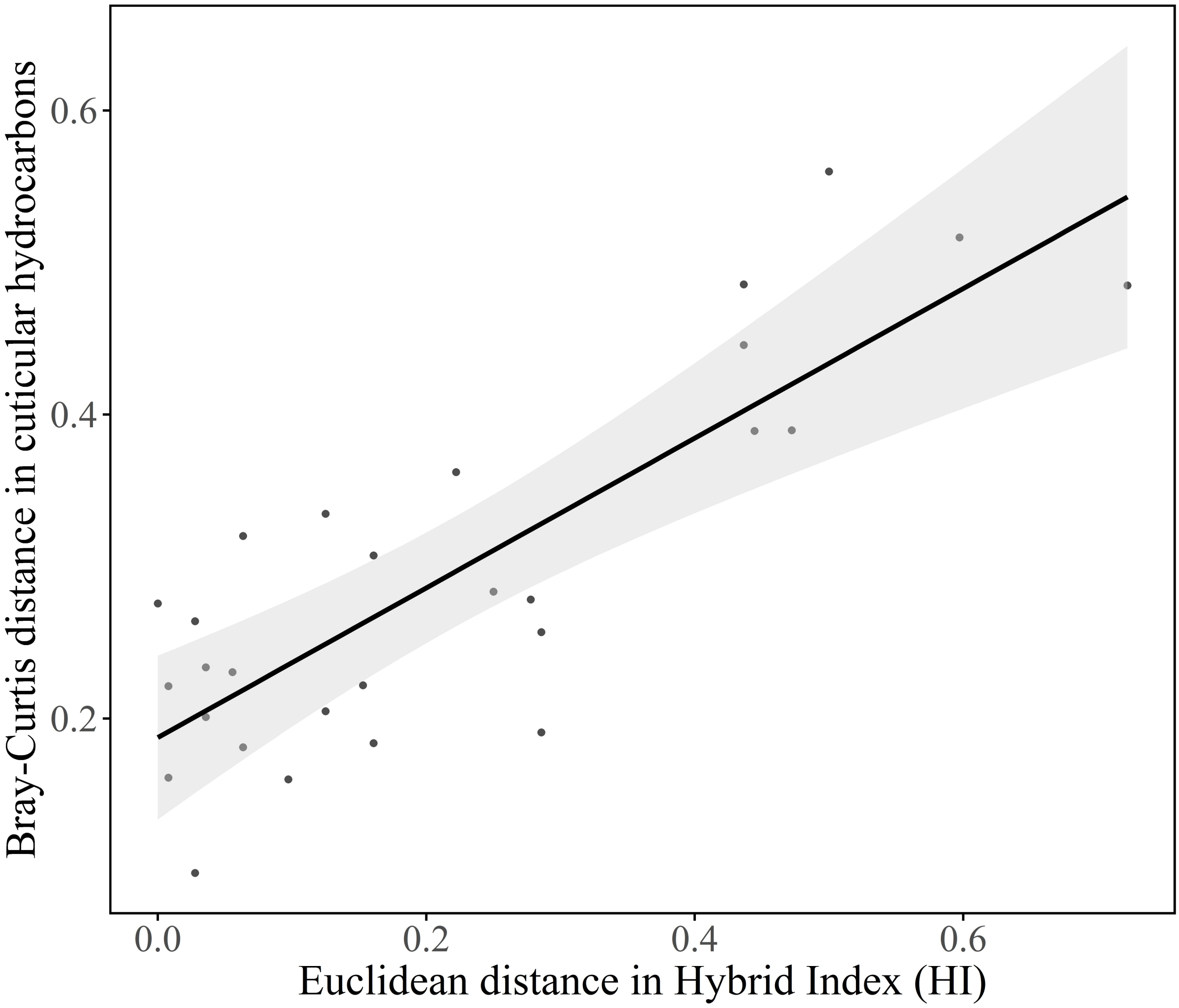

Figure S5. Positive correlation between the Bray-Curtis distance in cuticular hydrocarbons of hybrids and the Euclidean distance in the hybrid indices of their colonies. Each dot represents one pair of colonies.
